# Development of a small compound that regulates the function of a maltodextrin-binding protein of *Streptococcus pyogenes* by multifaceted screenings

**DOI:** 10.1038/s41598-025-02175-9

**Published:** 2025-06-02

**Authors:** Tsukushi Yamawaki, Makoto Nakakido, Satoru Nagatoishi, Jose M. M. Caaveiro, Daisuke Kuroda, Chihiro Aikawa, Ichiro Nakagawa, Kouhei Tsumoto

**Affiliations:** 1 Department of Chemistry and Biotechnology, School of Engineering, The University of Tokyo, 7-3-1 Hongo, Bunkyo-Ku, Tokyo 113-8656 Japan; 2 Department of Bioengineering, School of Engineering, The University of Tokyo, 7-3-1 Hongo, Bunkyo-Ku, Tokyo 113-8656 Japan; 3 Medical Device Development and Regulation Research Center, School of Engineering, The University of Tokyo, 7-3-1 Hongo, Bunkyo-Ku, Tokyo 113-8656 Japan; 4 Department of Protein Drug Discovery, Graduate School of Pharmaceutical Sciences, Kyushu University, 3-1-1 Maidashi, Higashi-Ku, Fukuoka, 812-8582 Japan; 5 Research Center for Drug and Vaccine Development, National Institute of Infectious Diseases, 1-23-1 Toyama, Shinjuku-Ku, Tokyo 162-8640 Japan; 6 Section of Applied Veterinary Sciences, Division of Veterinary Sciences, Department of Veterinary Medicine, Obihiro University of Agriculture and Veterinary Medicine, Hokkaido, 080-8555 Japan; 7 Department of Microbiology, Graduate School of Medicine, Kyoto University, Yoshida-Konoe-Cho, Sakyo-Ku, Kyoto, 606-8501 Japan; 8 Medical Proteomics Laboratory, The Institute of Medical Science, The University of Tokyo, Minato-Ku, Tokyo 108-8639 Japan

**Keywords:** *Streptococcus pyogenes*, Maltose/maltodextrin-binding protein, Small compound, Target based screening, Antimicrobial resistance, Accessibility, Biochemistry, Biophysics, Drug discovery, Microbiology, Molecular medicine

## Abstract

Group A *Streptococcus* (GAS) are gram-positive bacteria that cause various symptoms. The treatment of GAS infections currently relies on antibiotics, but new treatment options are needed due to the spread of antibiotic resistance. To develop novel treatment methods that circumvent the generation of antibiotic resistance, we used virtual screening followed by several biophysical-based screening methods to identify antibacterial compounds that target SPs0871, which is a maltodextrin-binding protein that is involved in carbohydrate catabolism in GAS. We narrowed down the list of compounds in the library via multi-step screening and finally isolated a compound that bacteriostatically inhibited the growth of GAS. Together with our previous study showing that an anti-SPs0871 variable heavy domain of heavy chain antibody, which completely blocked ligand binding, did not suppress bacterial growth, our results provide guidelines for designing an antistreptococcal therapeutic.

## Introduction

Group A *Streptococcus* (GAS) are gram-positive bacteria that cause a variety of superficial, sequelae, and invasive symptoms^1^. The treatment of GAS infections currently relies predominantly on antibiotics. However, due to the overuse of antibiotics, antibiotic resistance among bacteria is spreading^1,2^. Moreover, GAS infections have been rapidly increasing after the COVID-19 pandemic^3^. Therefore, new treatment options are sorely needed. Although vaccines against GAS are being investigated, none have been licensed to date^4^. Other therapies, such as using bacteriophages and nanoparticles, have also been investigated, but challenges remain in their use^5^.

Generally, two main types of drug discovery methods exist for compound screening: phenotype-based screening and target-based screening^6^. Phenotype-based screening has been widely adopted, in which hit candidates are selected based on the phenotype when compounds are added to bacterial cells. However, this strategy requires the target deconvolution process to identify the target molecules^7^. On the other hand, target-based screening, in which hit candidates are selected using pre-determined target molecules, can be customized based on the molecular mechanism of a drug effect on the target molecule at an earlier stage of multi-step screenings. Therefore, target-based screening to develop therapeutic compounds with high specificity is generally faster, easier, and cheaper than phenotype-based screening^8^. Indeed, Croston et al. showed that target-based screening has advantages in drug discovery for human diseases such as cancer and autoimmune disease, as it can identify a variety of genes closely associated with a disease^8^.

In recent years, researchers have focused on developing antibacterial drugs that target virulence factors to reduce selective pressure and circumvent the generation of antibiotic resistance^9^. Among the virulence factors in GAS that can be targeted, proteins involved in complex carbohydrate catabolism have been implicated in pathogen infectivity^10^. SPs0871 is a maltodextrin-binding protein that is part of the maltodextrin transport system^11^. Shelburne et al. previously showed that the knockout of the maltodextrin-binding protein gene did not suppress streptococcal growth in a nutrient-rich medium and that the protein played an essential role in bacterial growth in human saliva^12^. Since maltodextrin is believed to enter the glycolytic system by being converted to glucose and β-Glc 1-P after uptake and further converted to glucose 6-phosphate in other bacterial species^13^, a similar metabolism is expected to occur in GAS. These results suggested that the inhibition of SPs0871 suppresses bacterial growth in a context-dependent manner and that it would be a promising virulence factor for therapeutic targeting.

We previously obtained a variable heavy domain of heavy chain (VHH) antibody against SPs0871 and found that it inhibited ligand binding in vitro, but it showed no effect in the bacterial growth assay^11^. We hypothesized that the observed lack of antibacterial activity was due to the inability of the antibody to access the target protein on the cell surface^14^. Therefore, we decided to screen for smaller compounds that can pass through the cell surface capsule as a modality to target SPs0871 and inhibit its function.

Multiple methods of target-based compound screening have been established. For example, computational techniques such as structure-based virtual screening, molecular docking, and molecular dynamics simulations are standard methods used in structure-based drug design^15^. High-throughput screening methods using recombinant proteins, which rely on biophysical techniques including surface plasmon resonance (SPR), mass spectrometry, and differential scanning fluorimetry (DSF), are also popular^16^. ATP-binding inhibitors are well-known small molecular inhibitors that competitively bind to the ligand binding site and inhibit the target’s function. In developing such inhibitors, targeting conformational changes in target proteins has attracted attention^17,18^. For example, Cheeseman et al. reported that heat shock protein 70 inhibitors bind to their target protein with high affinity due to conformational changes in the protein^18^. Additionally, Homburg et al. showed that a maltodextrin-binding protein changed the conformation upon ligand binding^19^. Based on these findings, we hypothesized that targeting a conformational change would be a promising approach to developing a functional inhibitor against the maltodextrin-binding protein.

In this study, we used target-based screening, as we believe that targeting specific proteins in bacteria can lead to developing effective antimicrobial agents. We first performed virtual screening for the two-state conformation of SPs0871. Subsequently, biophysical-based screenings using SPR and DSF were carried out to narrow down the list of hit compounds. Finally, we evaluated the antibacterial activity of the hit compounds against GAS.

## Results

### Crystal structure and physicochemical analysis of SPs0871

**Fig. 1:**
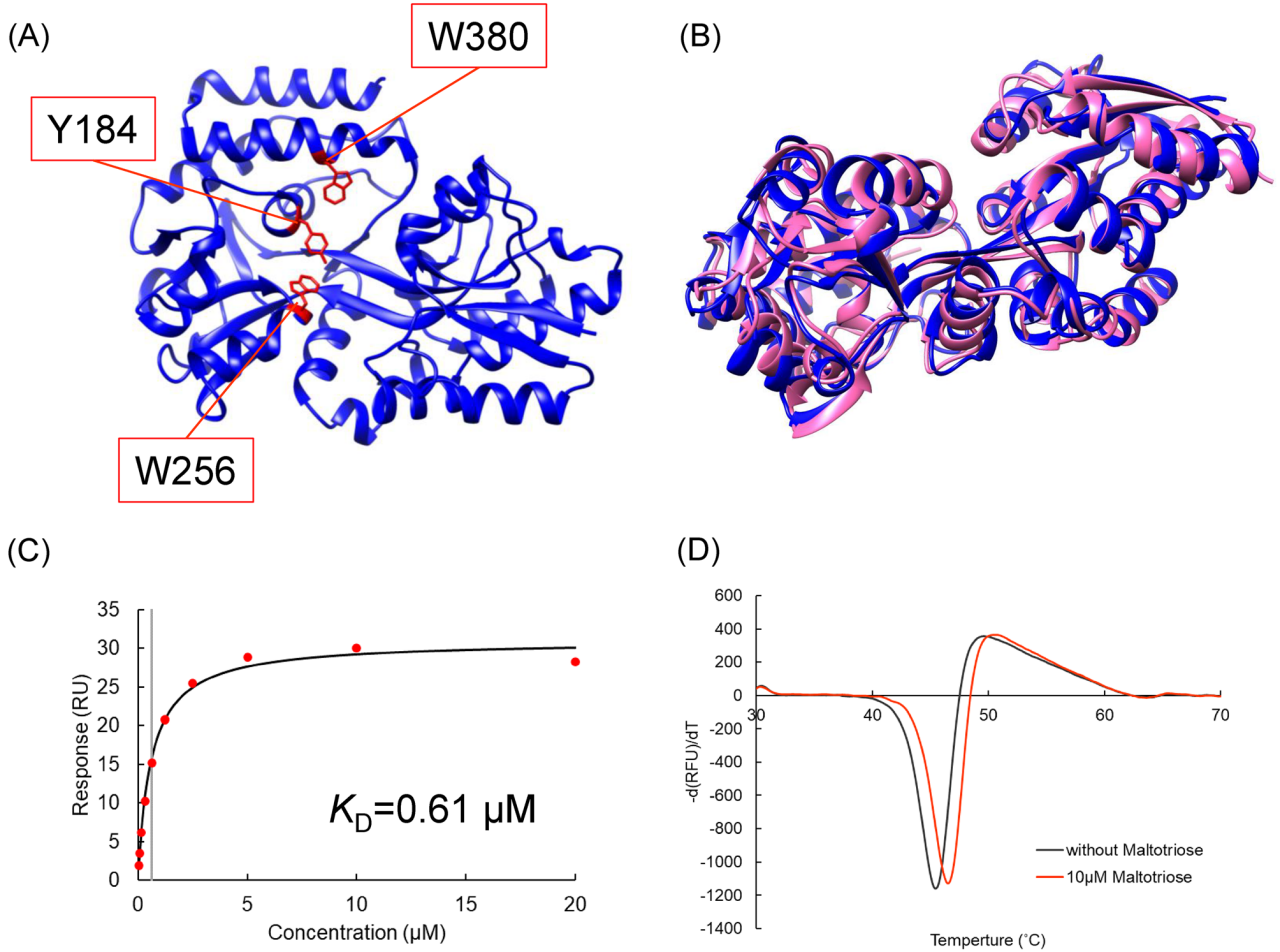
Crystal structure and physicochemical analysis of SPs0871. (**A**) Structure of SPs0871. Three aromatic residues that are important for ligand binding are shown in red. The figure was created using UCSF chimera version 1.16. (**B**) Overlay of SPs0871 (blue) and MalE1 (pink) derived from *L. casei* (Protein DataBank (PDB) ID: 5MKB). The figure was created using UCSF chimera version 1.16. (**C**) SPR analysis of the interaction between SPs0871 and maltotriose. (**D**) The melting temperature of SPs0871 assessed by DSF.

The crystal structure of SPs0871 in the absence of a ligand was determined at 2.20 Å (Table 1). Three aromatic residues critical for ligand binding (Y184, W256, and W380) lined one side of the binding pocket, similar to the structure of other maltodextrin-binding proteins (Fig. 1A)^19,20^. Superimposition of this crystal structure with that of maltodextrin-binding protein (MalE1) from *Lactobacillus casei* also in the absence of ligand (PDB ID: 5MKB) revealed high sequence homology and overlapping structures (Fig. 1B). The amino acid residues structurally corresponding to the ligand binding site of SPs0871 in maltodextrin binding proteins from several bacteria including MalE1 are summarized in Table 2. The 6 of 16 residues in MalE1 that were identified in previous studies as being involved in ligand interactions are conserved in SPs0871^19^. Given that other maltodextrin-binding proteins have been reported to adopt a closed conformation upon sugar binding, SPs0871 is likely to undergo a similar conformational change^19^.

**Table 1:** Data collection and refinement statistics.

Data collection	SPs0871 unbound
Space Group	H3 2
Unit cell	
a, b, c (Å)	118.2, 118.2, 353.9
α, β, γ (°)	90.0, 90.0, 120.0
Resolution (Å)	45.3–2.20 (2.32–2.20)
Wavelength	1.0000
Observations	832,407 (83,832)
Unique reflections	47,995 (6,275)
*R*_*merge*_	0.084 (0.352)
*R*_*p.i.m*_	0.020 (0.098)
CC_1/2_	0.999 (0.985)
*I / σ (I)*	21.9 (6.8)
Multiplicity	17.3 (13.4)
Completeness (%)	98.4 (89.4)
Refinement Statistics	
Resolution (Å)	45.3–2.20
*R*_*work*_ / *R*_*free*_ (%)	18.5 / 22.4
No. copies	2
No. atoms	
Protein	5,642
Water	312
B-factor (Å^2^)	
Protein	43.2
Water	35.6
Ramachandran Plot	
Preferred (%)	95.7
Allowed (%)	4.3
Outliers (%)	0.0
RMSD Bond (Å)	0.005
RMSD Angle (°)	1.46
PDB entry code	9KHA

Statistical values in parenthesis refer to the highest resolution bin.

**Table 2:**
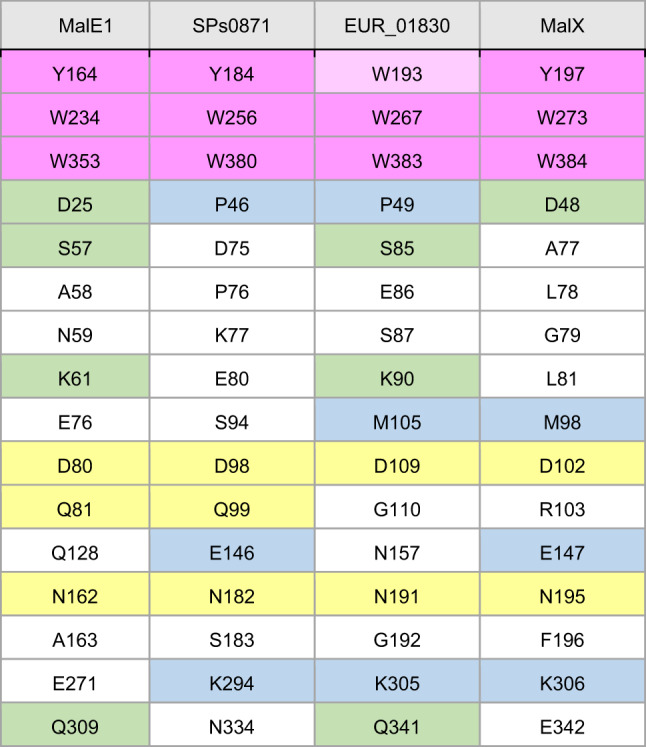
Comparison of residues interacting with the ligand in maltdextrin binding proteins. Conserved residues among more than 2 bacterial species are highlighted with blue, green, and yellow colors. Also, three residues important for ligand binding highlighted in pink.

Prior to small compound screening, we analyzed the ligand binding of SPs0871 quantitatively by SPR and its effect on thermal stability by DSF. SPR results showed a concentration-dependent increase in the response to maltotriose. The dissociation constant (*K*_D_) value was calculated to be 0.61 µM, which is close to the value we previously calculated using isothermal titration calorimetry (ITC) (Fig. 1C)^11^. In DSF, the presence of 10 µM maltotriose increased the melting temperature of SPs0871 by 1.2 °C (Fig. 1D). Although the melting temperature (T_m_) change was smaller than the value we previously reported, the difference was due to the maltotriose concentration^11^. We also confirmed that the T_m_ value increased in a sugar concentration-dependent manner (Fig. S1). These results illustrate that SPR- and DSF-based screening methods are applicable for studying the target protein SPs0871.

### Virtual screening and biophysical screening

**Fig. 2:**
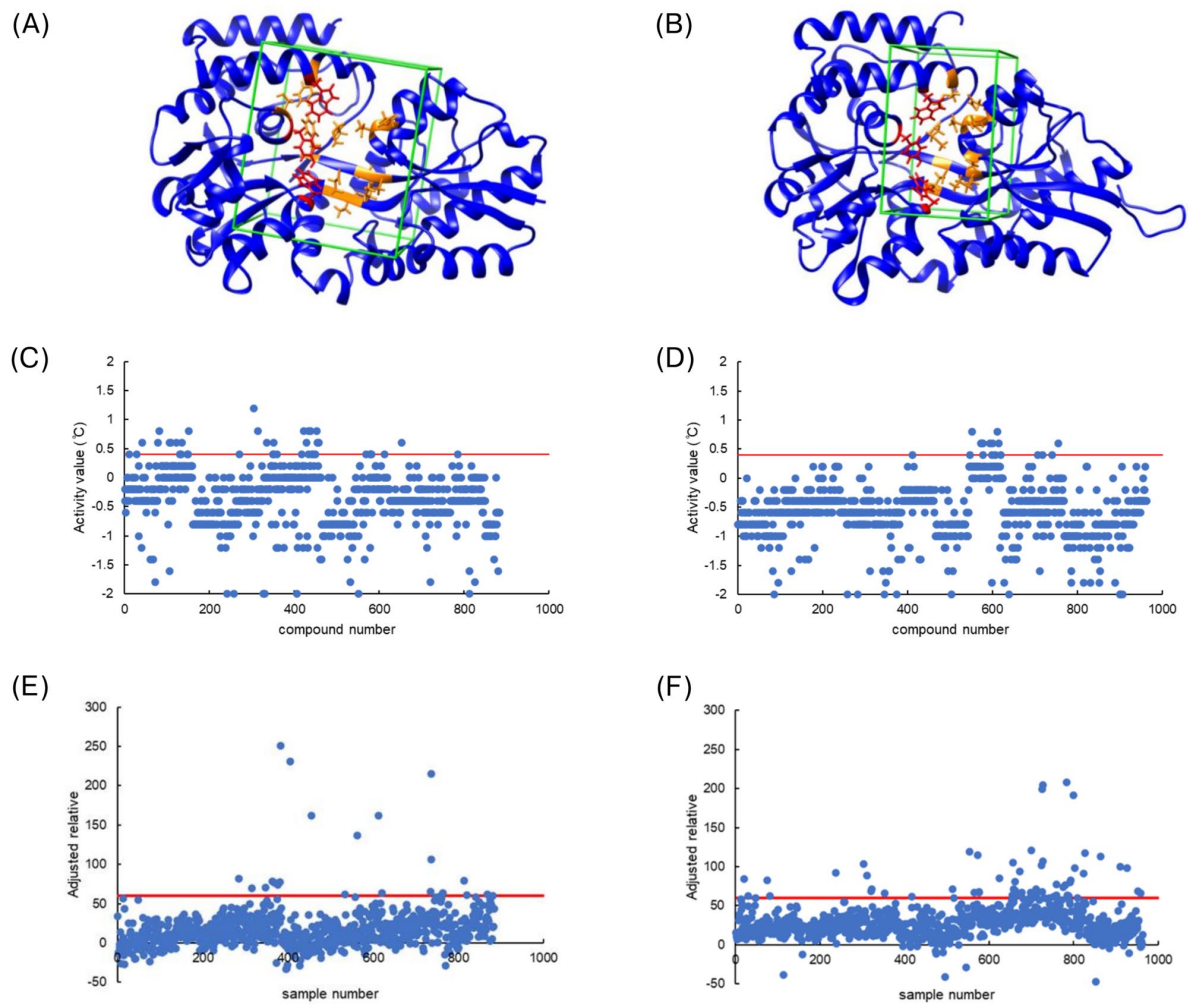
Screening results for SPs0871. (**A**–**B**) Docking conditions with the unbound form (**A**) and sugar-bound form (**B**); the side chains of the orange residues and compounds move within the grid box. The figures were created using UCSF chimera version 1.16. (**C**–**D**) DSF screening of the unbound form hit library (**C**) and sugar-bound form hit library (**D**). (**E**,**F**) SPR screening of the unbound form hit library (**E**) and sugar-bound form hit library (**F**). For (**C**–**F**), the red lines indicate the criteria for the respective screening.

Virtual screening was performed on compounds with molecular weight ranging from 300 to 700 from the Drug Discovery Initiative library at The University of Tokyo using AutoDock Vina (1.1.2)^21^. Because AutoDock Vina does not account for target protein backbone flexibility, we employed two structures for docking: the crystal structure obtained in this study, representing the “unbound form” of SPs0871, and a homology-modeled structure based on MalE1 from *L. casei* in complex with maltotriose (PDB ID: 5M28), representing the “sugar-bound form” of SPs0871 (Fig. 2A,B). Using both conformations allowed us to assess whether potential ligands induce conformational changes in SPs0871 upon binding. Although other alternative conformations may exist, we limited our analysis to these two states to reduce the search space for docking.

Before the virtual screening, we docked maltotriose to the bound form of SPs0871 to evaluate the stability of three key aromatic residues (Y184, W256, and W380) essential for ligand binding^19^. Allowing the side chains of these residues to remain flexible produced binding modes inconsistent with those observed in the crystal structure of MalE1 bound to maltotriose (PDB ID: 5M28). To resolve this issue, we fixed the orientations of these three aromatic residues while keeping the side chains of other residues around the binding pocket flexible. Under these conditions, we performed virtual screening using 141,675 compounds, and we selected 884 compounds for the unbound form (criteria ≦–9.8 kcal/mol) and 963 compounds for the sugar-bound form (criteria was ≦–11.9 kcal/mol) as hit compounds. Two hundred twenty-nine compounds were common to both forms, resulting in 1618 compounds for subsequent screening.

In the subsequent DSF screening, we measured the T_m_ value of SPs0871 in the presence of each compound. We also evaluated the structural stabilizing effect of each compound by binding it to SPs0871, which reflected the binding of the compound in the ligand binding pocket and the intramolecular interaction network. We included SPs0871 without small compounds as a negative control in each screening plate. The difference between the T_m_ value for each compound and the highest negative control T_m_ value in the same plate was used as the activity value for each compound. We defined compounds with activity values > 0.4 °C as hit compounds, which resulted in 59 compounds classified as hits (Fig. 2C,D).

In parallel, we conducted SPR screening to evaluate the direct binding of compounds to SPs0871. We immobilized SPs0871 on a sensor chip, flowed each compound to the chip in sequence, and evaluated the response for each compound. The response derived from maltotriose binding was designated as the positive control, with a relative value 100, and the response derived from the injection of running buffer was designated as the negative control, with a relative value 0. The molecular weight corrected value for each compound was used as the activity value. We defined hit compounds as those with an activity value ≥ 60, which resulted in 81 compounds classified as hits (Fig. 2E,F).

Collectively, three hit compounds were detected in both DSF and SPR screenings. In addition to these hit compounds, we chose one compound with a high activity value (1.2 °C) in the DSF screening and three compounds with a high activity value (> 80) in the SPR screening with no negative activity value in the DSF screening as additional hit compounds. In total, seven compounds were selected as hits from SPR and DSF screening (Table 3, Fig. S2).

**Table 3:**
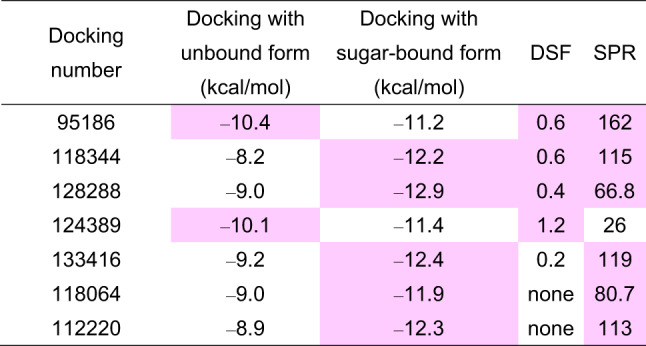
Screening hits. The values that met the inclusion criteria are shown in pink.

**Fig. 3:**
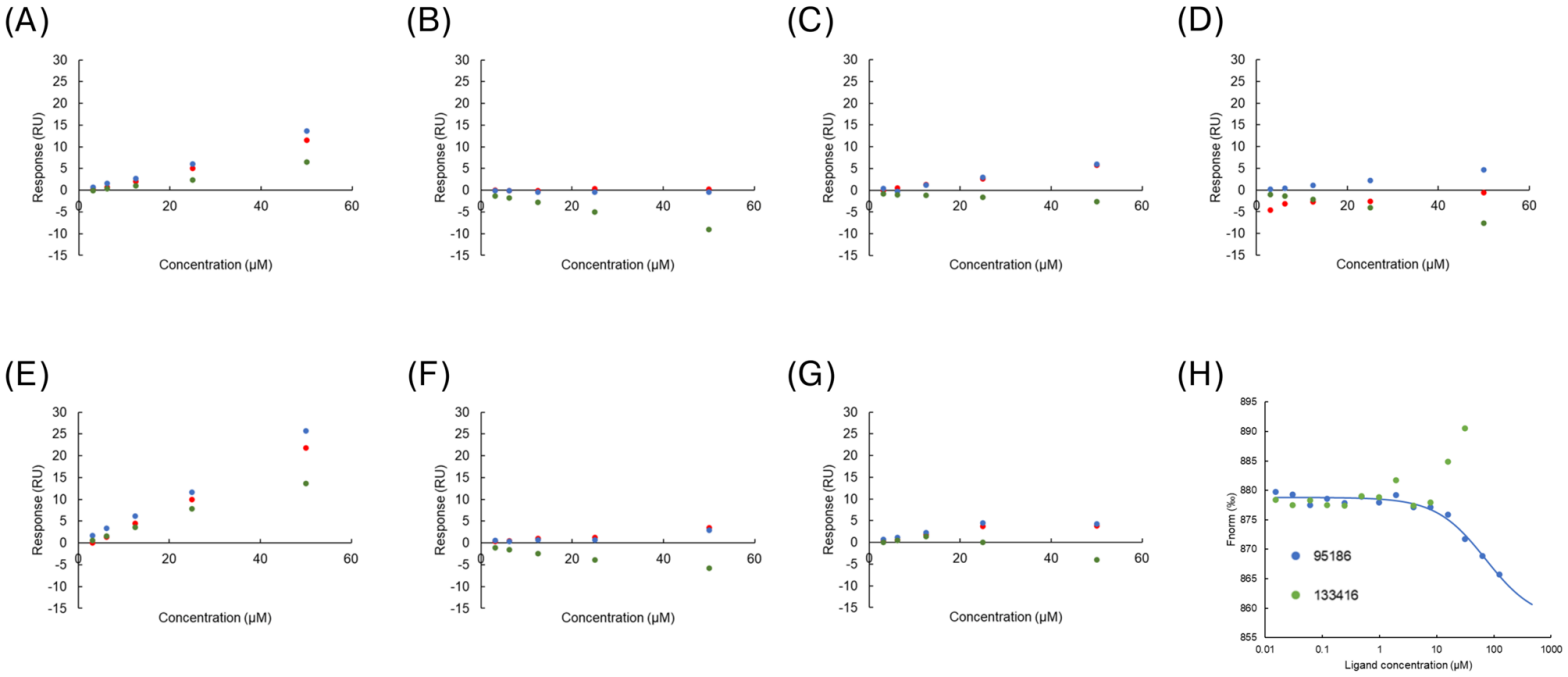
Concentration-dependent analysis. (**A**–**G**) Concentration-dependent analysis by SPR. (**A**) 95,186. (**B**) 118,344. (**C**) 128,288. (**D**) 124,389. (**E**) 133,416. (**F**) 118,064. (**G**) 112,220. (**H**) Concentration-dependent analysis by MST.

We then checked the concentration dependence of the binding of the seven compounds using SPR (Fig. 3A–G). Three independent experiments were conducted for each compound, and the concentration-dependent increase in response was reproducibly observed only for compounds 95,186 and 133,416. We then analyzed the binding of these compounds using another biophysical technique, microscale thermophoresis (MST), and only compound 95,186 showed a concentration-dependent response (Fig. 3H). Based on the DSF, SPR, and MST screening results, we chose 95,186 as the hit compound and proceeded with molecular characterization of this compound.

### Confirmation of 95,186 binding to the ligand binding pocket of SPs0871

**Fig. 4:**
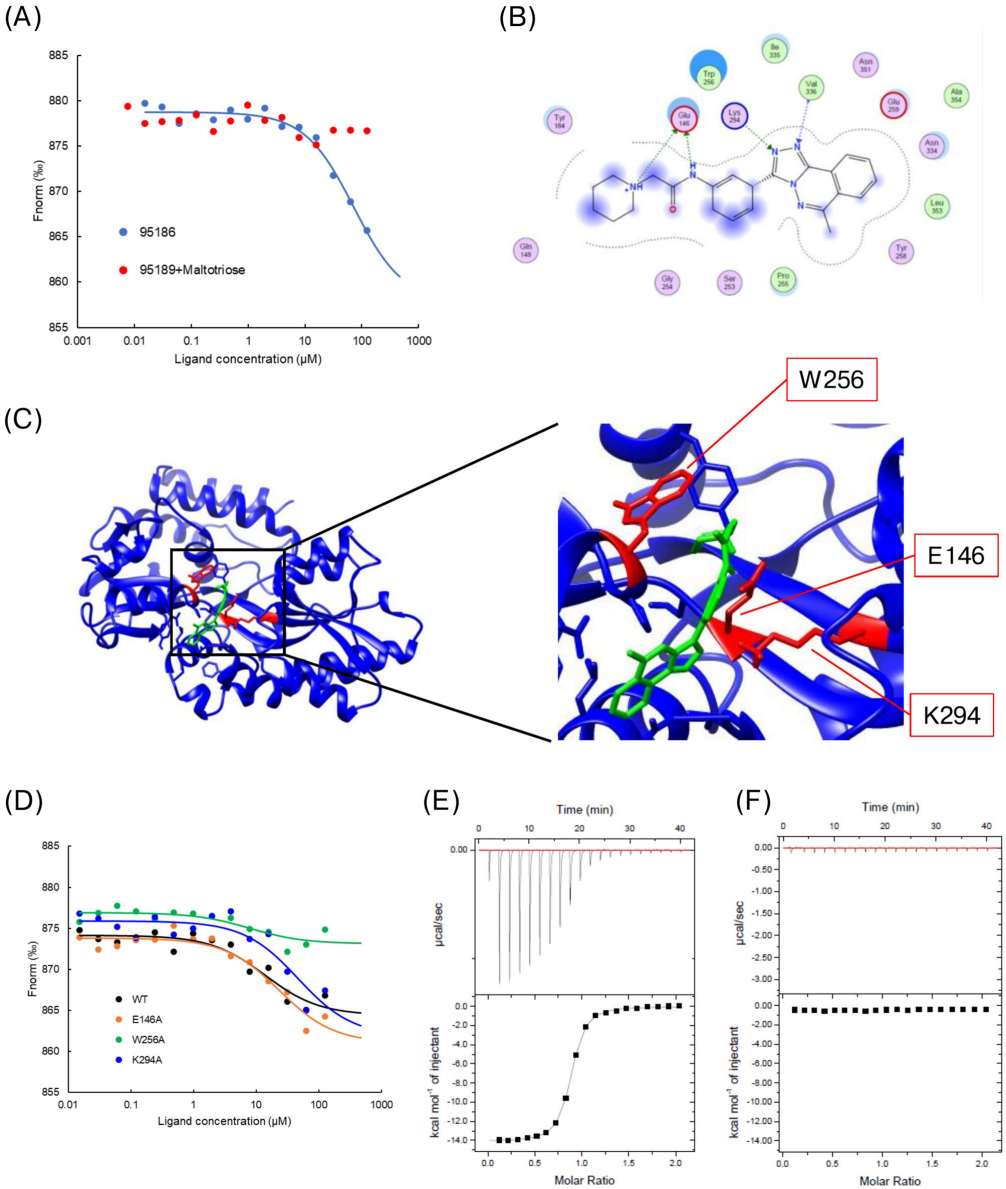
Competition between 95,186 and the ligand. (**A**) Competition between 95,186 and maltotriose by MST. (**B**) 2D diagram of binding sites predicted by Glide. (**C**) Overall figure of binding by predicted by Glide. The figure was created using UCSF chimera version 1.16. (**D**) Confirmation of 95,186 binding to mutants of SPs0871 by MST. (**E**,**F**) Titration curve of the ITC analysis of the interaction between maltotriose and (**E**) SPs0871 wild type and (**F**) SPs0871 W256A (alanine mutant).

To assess whether 95,186 competes with the natural ligand maltotriose in binding to SPs0871, we conducted MST analysis in the presence or absence of maltotriose. In the presence of maltotriose, the 95,186-derived concentration-dependent response disappeared, indicating that 95,186 bound to the ligand binding pocket and competed with the ligand (Fig. 4A). When we performed additional docking calculations using the Glide docking program (Schrodinger, New York, NY, USA) to identify hotspot residues for the compound binding^22^, E146 and K294 were predicted to form hydrogen bonds and W256 was predicted to form van der Waals interactions during compound binding (Fig. 4B,C). We validated these predictions using recombinant SPs0871 mutants in which these residues were mutated to alanine. Only the W256A mutation attenuated the response change upon the binding (Fig. 4D). Given that W256 is an essential residue for ligand binding (Fig. 4E–F), this result is consistent with the result shown in Fig. 4A, which suggested competition between 95,186 and the ligand.

### Analysis of compound derivatives

**Fig. 5:**
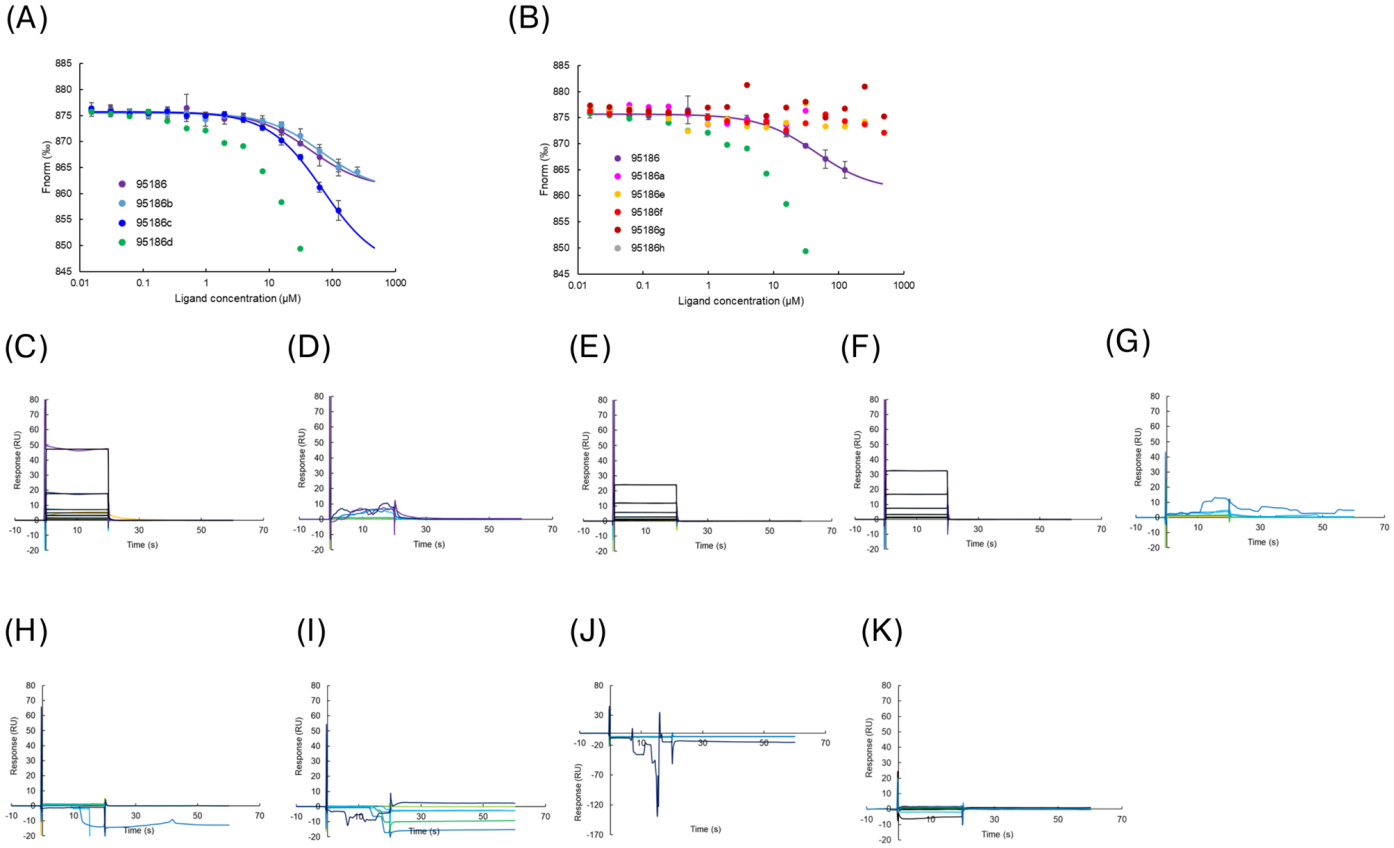
Analysis of 95,186 derivatives (**A**,**B**) Concentration-dependent analysis by MST. (**C**–**K**) Concentration-dependent analysis by SPR. (**C**) 95,186. (**D**) 95186a. (**E**) 95186b. (**F**) 95186c. (**G**) 95186d. (**H**) 95186e. (**I**) 95186f. (**J**) 95186 g. (**K**) 95186 h.

To increase the affinity of 95,186 for SPs0871, we assessed the binding of eight 95,186 derivatives in which mainly piperidine (95186b-g) and 1,2,4-triazolo[3,4-a]phthalazine (95186 h) were changed (Table 4). MST results showed a concentration-dependent response change for 95186b, 95186c, and 95186d but no change for 95186a, 95186e, 95186f., 95186 g, and 95186 h (Fig. 5A,B). Subsequently, we performed SPR for cross-validation and observed concentration-dependent responses for 95186b and 95186c but not for 95186d-h (Fig. 5C–K). These results suggested that 1,2,4-triazolo[3,4-a]phthalazine is essential for 95,186 binding and that piperidine is preferred for the opposite end. The *K*_D_ values calculated by MST analysis for the compounds that showed a binding response in both MST and SPR were 44.7, 67.1, and 71.7 µM for 95,186, 95186b, and 95186c, respectively.

**Table 4:**
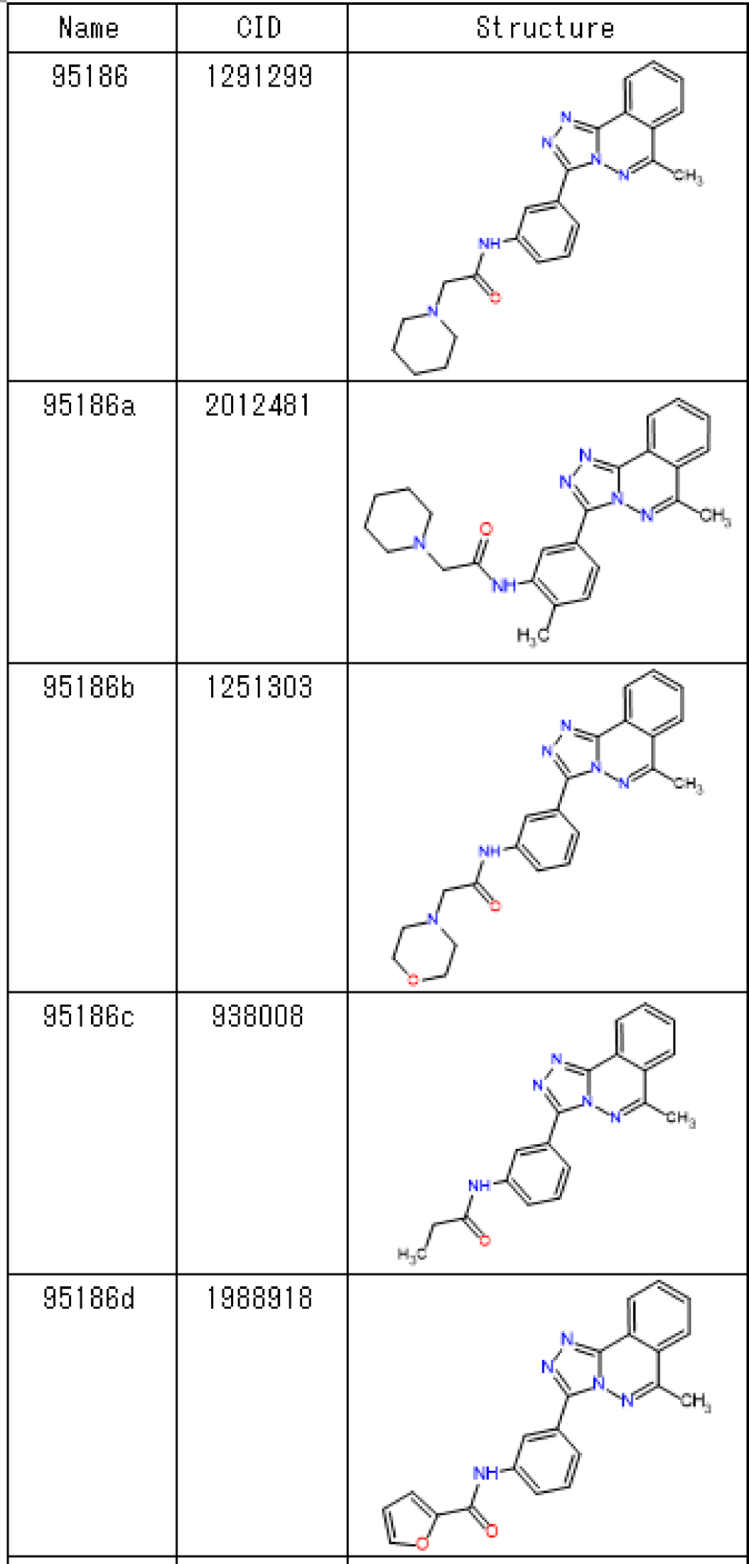

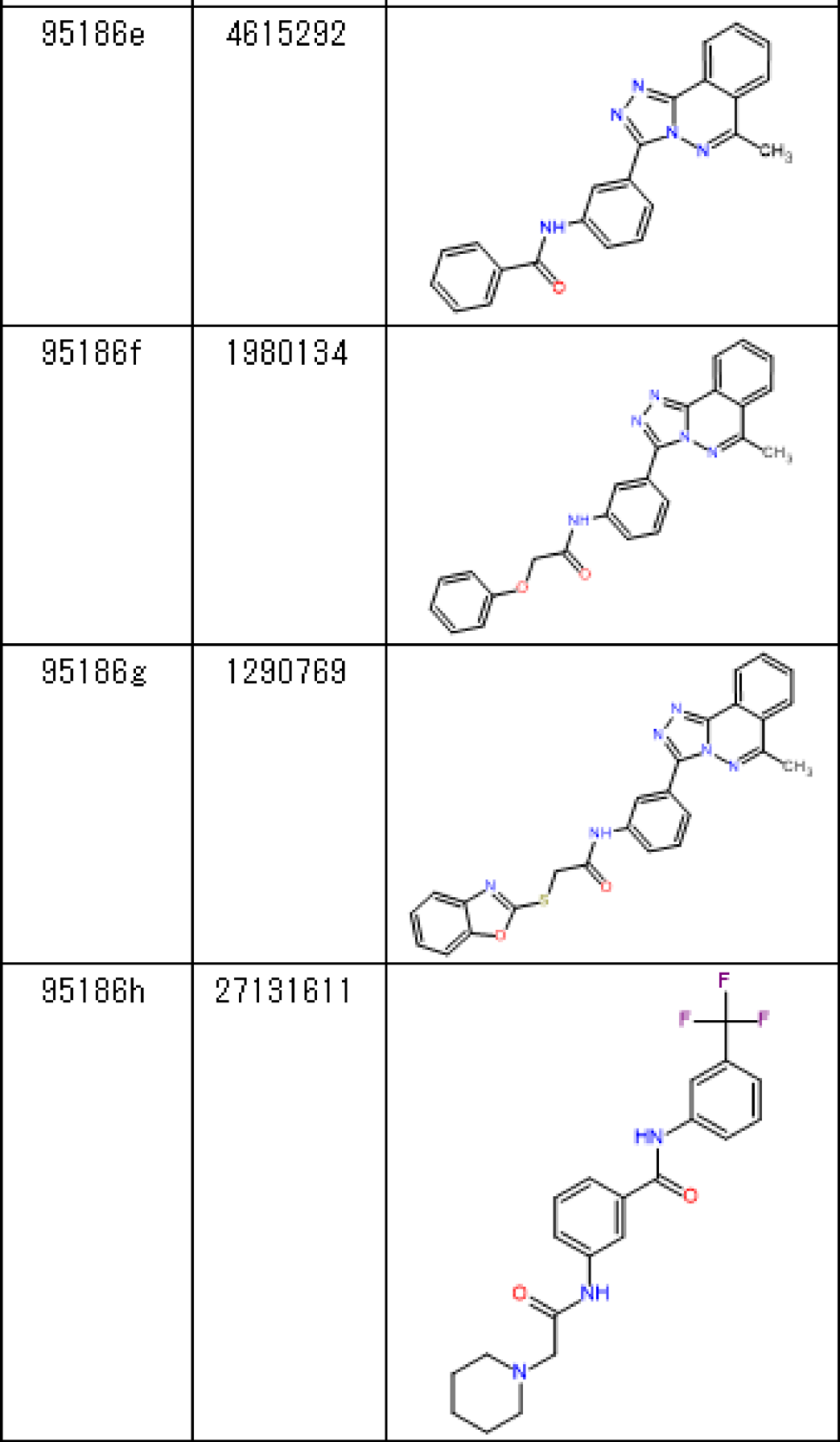
95186 derivatives.

### Inhibition of GAS growth by 95,186 compounds

**Fig. 6:**
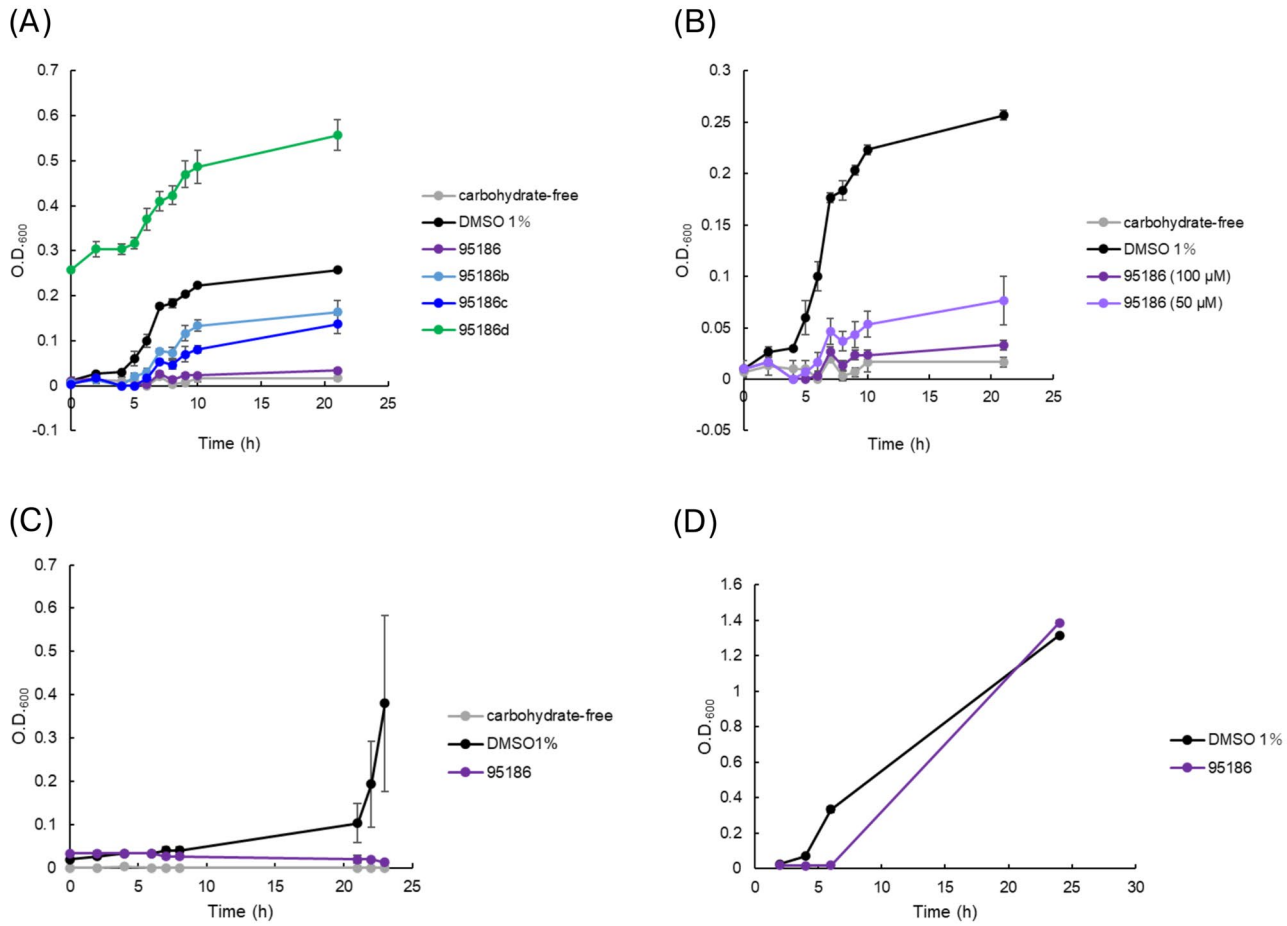
Growth assay. (**A**) JRS4 strain growth assay in maltotetraose medium. (**B**) Concentration-dependent effects of 95,186. (**C**) SSI-1 strain growth assay in CDM medium. (**D**) JRS4 strain growth assay of compound 95,186 in THY medium after the assay in maltotetraose medium. The data are means ± standard deviations for triplicate experiments.

To assess whether the compounds that showed a response change in MST or SPR are effective against bacteria, we performed a growth assay using experimental GAS strain *S. pyogenes* strain JRS4. Bacteria were incubated in a carbohydrate-free chemically defined medium (CDM) supplemented with maltotetraose, which is thought to be taken up only via the SPs0871 pathway^23^. The three compounds at 100 µM that showed a concentration-dependent response in SPR analysis (95,186, 95186b, and 95186c) suppressed GAS growth (Fig. 6A). The initial optical density at 600 nm (OD_600_) values for 95186d were high because of aggregation of the compounds in the medium as well as bacterial growth. Intriguingly, 95,186 had the highest growth inhibition potential, which correlated with the magnitude of the SPR response and the *K*_D_ values calculated from MST analysis. The growth suppression effect by 95,186 was concentration dependent above the *K*_D_ value concentration, supporting the effect would be specific to targeting pathway (Fig. 6B). Further, we also evaluated the antibacterial effect of 95,186 against SSI-1 strain, which is isolated from a STSS patient^24^. We confirmed that the compound also suppressed the maltodexitrin dependent growth of SSI-1 (Fig. 6C). We then transferred JRS4 exposed to 95,186, which showed growth inhibition, into THY (Todd-Hewitt Broth w/ 0.2% Yeast Extract) medium to determine if the bacterial suppression effect was bactericidal or bacteriostatic. Bacterial growth was observed, suggesting that the growth inhibition by the compound was due to bacteriostatic action (Fig. 6D).

### Evaluation of bacterial species specificity of the 95,186 compounds

**Fig. 7:**
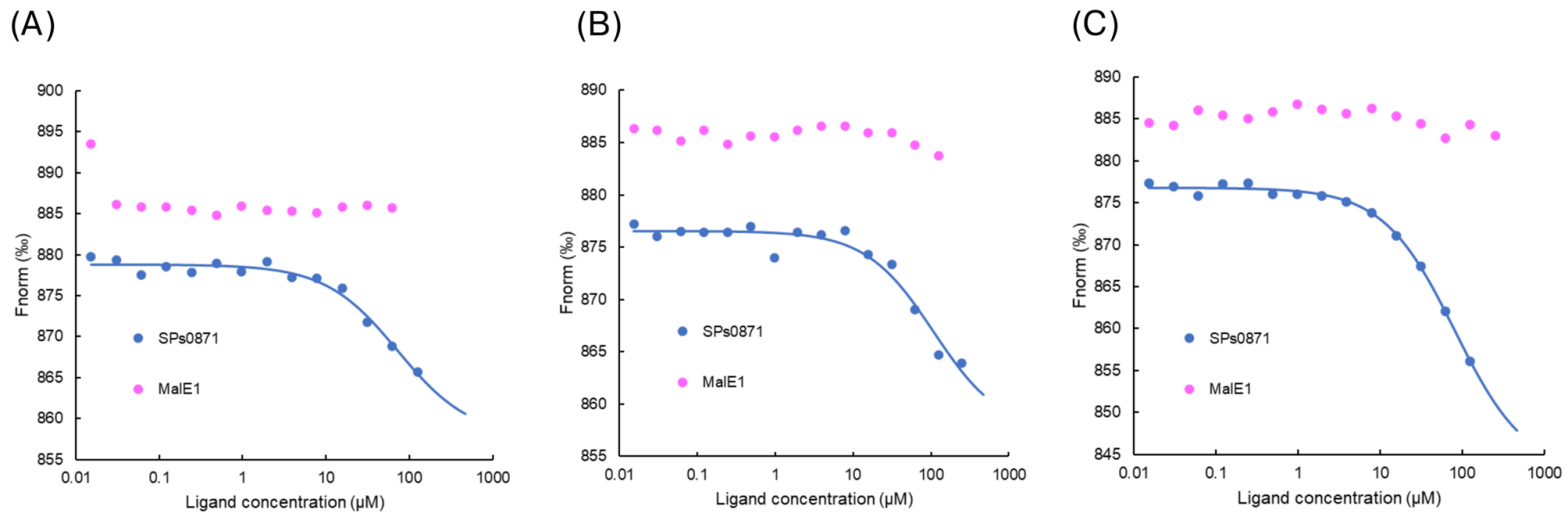
MST analysis of bacterial species specificity of compound 95,186 and derivatives. (**A**) 95,186. (**B**) 95186b. (**C**) 95186c.

Finally, to gain an insight into the specificity of the three compounds for bacterial species, we prepared MalE1 from *L. casei* as a recombinant protein and used MST to assess the binding of compounds 95,186, 95186b, and 95186c to MalE1. No concentration-dependent response changes were detected for either compound (Fig. 7A–C), suggesting that the hit compounds had bacterial species specificity.

## Discussion

This study successfully obtained a hit compound (95,186) that suppresses GAS growth via bacteriostatic action. Although bactericidal action appears required to treat GAS infection, compounds with bacteriostatic activity would also be relevant for clinical usage. Indeed, many bacteriostatic agents, including chloramphenicol, clindamycin, and linezolid, have been effectively used to treat infectious diseases^25^. Several studies have generated antibacterial compounds against GAS through target-based screening^26,27^, but they all target secreted factors. As previously described, targeting bacterial membrane proteins remains challenging due to the inaccessibility of the target protein^14^. Indeed, we previously identified a VHH that inhibited maltodextrin binding but did not inhibit GAS growth^11^. In the current study, we isolated hit compounds targeting membrane-associated protein SPs0871 and identified a compound that suppressed bacterial growth. Taken together with a previous study showing that maltodextrin utilization is critical for GAS growth in saliva where the glucose levels are very low^12,28^, it is likely that the functional inhibition of SP0871 by the compound prevented maltodextrin uptake in CDM, leading to the depletion of enough amount of glucose produced from the maltodextrin thorough glycolytic pathway as energy source, and thereby suppressed the bacterial growth. To our knowledge, this is the first study to generate a small compound that targets membrane proteins on GAS and possesses antibacterial activity. Since an antibody targeting an antigen on the cell membrane of other gram-positive bacteria has been effective in bacterial assays^29^, difficulty accessing the membrane protein on GAS may be caused by the presence of the capsule, a highly developed defense mechanism of GAS^30^. The compound identified in our study has the appropriate molecular properties to break through the capsule. In addition, as SPs0871 is classified as a conditionally beneficial virulence factor for GAS, treatment with our compound would generate a weaker selection for resistance^9^. In summary, the compound identified in this study is an unprecedented type of GAS inhibitor.

Maltodextrin-binding proteins are thought to exist in the unbound conformation without ligands, and ligand binding drives the protein into the sugar-bound form^31^. Virtual screening with the two conformations followed by in vitro screening yielded a hit compound (95,186) that competed with the ligand and was derived from the hit library for the unbound form. Therefore, it is likely that 95,186 favorably binds to the unbound form. The docking score determined using AutoDock Vina between the unbound form and maltotriose was not high (–6.1 kcal/mol), and the initial binding to the unbound form may be more favorable for 95,186 than for maltotriose. Given that SPs0871 changes the conformation upon the ligand binding, the rapid binding and dissociation of the compound would suppress the conformational change of SPs0871 and thereby inhibit the ligand binding regardless of the large difference of apparent affinity. This scenario is supported by the growth inhibition caused by 95,186, even in a high concentration of maltodextrin, whose binding affinity to SPs0871 was higher than that of compound 95,186. In docking, the hit compounds from virtual screening for the sugar-bound form scored better than maltotriose (–7.7 kcal/mol), but no-hit compounds emerged from the library. This result was probably because the compounds could not induce conformational change to sugar-bound form as maltodextrin does.

We conducted small molecule screening using several biophysical methods to obtain hit compounds. SPR is capable of sensitive detection of binders over a wide concentration range. Additionally, the T_m_ shift in DSF indicates an enhancement of the intramolecular interaction network due to the binding of the compound to the protein, which may have implications for the attractive binding mode of the compound to the protein. In MST measurements, the change in the fluorescence signal is closely related to changes in surface charge and hydration state, which suggest changes in the physical properties of the protein associated with compound binding. We applied multiple biophysical methods to select compounds closely intertwined with the unique structural and physical properties of the target protein SPs0871. We also used this approach to evaluate bacterial species specificity by comparing the binding of the compounds 95,186 and its derivatives 95186b and 95186c to SPs0871 versus MalE1, a homolog protein derived from *L. casei*. Given that the compounds did not bind to MalE1, which possess high structural similarity with SPs0871 (Fig. 1B) and the residues involved in the ligand binding are not highly conserved among bacterial species (Table 2), the hit compounds would have bacterial species specificity.

In general, the potency of small molecule inhibitors is aimed at working at the nanomolar range^32^. In each of the experiments in this study, efficacy was confirmed in the range of tens to hundreds of micromoles, and further affinity improvement is needed for practical application. We plan to conduct further analyses of other derivatives and structural optimization based on the structure of the compound–SPs0871 complex. We believe that our structure–activity relationship information can be used effectively to improve antibacterial activity. It should be noted that the results of mutation analysis shown in Fig. 4 validated W256 as a binding hot spot, indicating that the compound binding region was successfully predicted. On the other hand, the binding of the compound to E146 and K294, which were also predicted as hot spot residues, were not validated by subsequent experiment. This result indicates that the predicted structure by docking may not be accurate and the orientation of the compound would be slightly different. The experimentally determined crystal structure of the SPs0871 in complex with the compound will allow more effective strategy to develop derivatives based on the accurate structural information. Nevertheless, considering the superior characteristics described above, our compound would provide a proof of concept toward the development of novel drug targeting GAS.

In summary, we used multifaceted screening methods to obtain a compound that competes with maltodextrin binding to SPs0871 and inhibits GAS growth. Our results provide essential insights for developing inhibitors as an antistreptococcal therapeutic.

## Methods

### Expression and purification of recombinant proteins

The SPs0871 (including mutants) gene fragment encoding amino acids 27–415 was cloned into *Escherichia coli* expression vector pCold I (for MST) or pCold SUMO, in which the His6-SUMO-tag was introduced into the pCold I vector (Takara, Shiga, Japan). The same process was performed for MalE1 derived from the *L. casei* gene fragment encoding amino acids 28–410. The recombinant protein was expressed and purified as previously described^11^.

### Compounds

Maltotriose and maltotetraose were purchased from Hayashibara Inc. (Okayama, Japan). The library used in the screenings and 95186 h were provided by the Drug Discovery Initiative library. The stock solution of all compounds was prepared in dimethylsulfoxide (DMSO) and stored at − 80 °C. Compounds 95,186, 95186a, 95186b, 95186c, 95186d, 95186e, 95186f., and 95186 g used in the hit validations were purchased from Pharmeks (Moscow, Russia) as powder and were dissolved in DMSO.

### Crystallization of SPs0871

Purified SPs0871 was dialyzed against 20 mM Tris pH 8.0. After dialysis, SPs0871 was concentrated to approximately 17 mg/mL. Crystallization was performed on an Oryx8 instrument (Douglas Instruments, Berkshire, UK) by mixing 0.5 µL of protein solution with solutions contained in commercial screening kits (Hampton Research, Aliso Viejo, CA, USA). Crystals were grown at 20 °C. A crystal of SPs0871 was obtained in a solution containing 1.8 M ammonium citrate tribasic pH 7.0. The crystal of SPs0871 was briefly incubated in mother liquor supplemented with 25% glycerol prior to flash-freezing in liquid nitrogen.

### Data collection and processing

Diffraction data were collected at beamline BL5A of the Photon Factory (Tsukuba, Japan) under cryogenic conditions (100 K). Diffraction images at a resolution of 2.20 Å were processed using the program MOSFLM^33^ and merged and scaled with the program SCALA^34^ of the CCP4 suite^35^. The structure of SPs0871 was determined by the method of molecular replacement using the program MOLREP^36^ and the structure of maltodextrin binding protein MalE1 from *Lactobacillus casei* without ligand as a model (PDB entry code 5MKB^19^). The structure was refined with the program REFMAC5^37^ and built manually with COOT^38^. Validation was carried out using PROCHECK^39^. Data collection and structure refinement statistics are given in Table 1. The coordinates and structure factors of SPs0871 have been deposited in the PDB with entry code 9KHA.

### SPR

The binding of maltotriose and compound derivatives to SPs0871 was assessed using a Biacore T200 instrument (Cytiva, Marlborough, MA, USA). SPs0871 was immobilized at 4300–4700 RU on the surface of a CM5 sensor chip (Cytiva) by the amine-coupling method according to the manufacturer’s instructions. The running buffer was phosphate buffered saline with Tween (PBS, 0.005% Tween 20), 5% DMSO. To measure the interaction of maltotriose with SPs0871, a range of concentrations of maltotriose (0.039–20 µM) were injected at a flow rate of 30 μL min^–1^ with a 20 s association time and a 40 s dissociation time. To measure the interaction of compound derivatives with SPs0871, a range of concentrations of compound derivatives (3.125–200 µM) were injected at a flow rate of 30 μL min^–1^ with a 20 s association time and a 40 s dissociation time. Data analysis was carried out using BIAevaluation software (Cytiva, version ver3.2.1, https://www.cytivalifesciences.com/ja/jp/support/software/biacore-downloads?_gl=1*18vz87l*_gcl_au*MjA3MjI1ODE4Ni4xNzQyOTU1NzY3*_ga*OTQ5NDM5MTMyLjE3NDI5NTU3Njg.*_ga_CS9H0CZBWW*MTc0NDQ0NjkwMy41LjEuMTc0NDQ0ODAwNC4wLjAuMA.). The *K*_D_s of the interactions were calculated from Scatchard plots.

### DSF

DSF was performed on a CFX Connect Real-Time System (Bio-Rad, Hercules, CA, USA). The buffer was 1 × PBS, 5% DMSO except an experiment to assess the change of T_m_ value in a sugar concentration-dependent manner (Figure S1), in which DMSO was not added into the buffer. In the initial screening, the final concentration of the compounds was 100 µM and the concentration of SPs0871 was a 2 μM. One thousandth of the sample volume of SYPRO Orange Protein Gel Stain (5000 × concentrated in DMSO; Invitrogen, Waltham, MA, USA) was added, and samples containing 5 × SYPRO Orange were loaded onto 96-well PCR Plates (Bio-Rad) for measurement. The denaturation of protein was observed via fluorescence emitted from SYPRO Orange upon absorption to the exposed hydrophobic surface.

### Virtual screening and docking refinement

Virtual screening was conducted using AutoDock Vina (1.1.2)^21^. A model structure for the sugar-bound form was prepared with MODELLER (10.1)^40^ based on MalE1 from *L. casei* in complex with maltotriose (PDB ID: 5M28). The following side chains surrounding the binding pocket were set as flexible: ASP98, GLN99, ILE145, GLU146, SER183, PHE290, VAL293, LYS294, MET370, and PHE379 for the unbound form and ASP75, GLN79, ASP98, GLN99, GLU146, LYS294, MET370, MET376 for the sugar-bound form. Docking was performed with a grid box that encompassed the ligand-binding pocket (Fig. 2A,B). We performed virtual screening using 141,675 compounds. For each compound, the best docking score was recorded as its activity value.

To explore hotspot residues, additional docking calculation was performed in Maestro using the Glide docking program^22^. A compound was built using “Maestro build panel” and optimized to lower energy conformers using Ligprep (Schrodinger, version 13.6.122, https://www.schrodinger.com/platform/products/ligprep/). A protein structure was prepared for docking using “protein preparation wizard” in Maestro. (Schrodinger, version 13.6.122, https://www.schrodinger.com/platform/products/maestro/). After preparation, the docking simulation was started, and the final evaluation of ligand–protein binding was based on the Glide score.

### SPR screening

Direct binding of compounds to SPs0871 was examined using a Biacore 8 K instrument (Cytiva). SPs0871 was immobilized at 3500–5300 RU on the surface of a CM5 sensor chip (Cytiva) by the amine-coupling method according to the manufacturer’s instructions. The running buffer was PBS-T (PBS, 0.005% Tween 20), 5% DMSO. In the screening, the final concentration of each compound was 100 µM, and samples were injected at a flow rate of 30 μL min^–1^ with a 30 s association time. To check the concentration dependency, a range of concentrations of the compounds (3.125–50 µM) were injected at a flow rate of 30 μL min^–1^ with a 20 s association time and a 30 s dissociation time. Data analysis was carried out using Biacore 8 K Evaluation software (Cytiva, ver3.0.12, https://www.cytivalifesciences.com/ja/jp/support/software/biacore-downloads?_gl=1*18vz87l*_gcl_au*MjA3MjI1ODE4Ni4xNzQyOTU1NzY3*_ga*OTQ5NDM5MTMyLjE3NDI5NTU3Njg.*_ga_CS9H0CZBWW*MTc0NDQ0NjkwMy41LjEuMTc0NDQ0ODAwNC4wLjAuMA.).

### MST

MST was performed on a Monolith NT.115 instrument using Monolith NT.115 Series, Premium Capillaries, and His-tagged SPs0871 was labeled using the Monolith His-Tag Labeling Kit RED-Tris-NTA 2nd Generation (NanoTemper Technologies GmbH, Munich, Germany). The final concentration of SPs0871 was 100 nM, and the final concentration of the dye was 5 nM. The buffer was PBS-T (PBS, 0.005% Tween 20), 5% DMSO. A range of concentrations of compounds (15.3 nM to 500 µM) was tested. Since the affinity of maltotriose calculated by MST was 6.2 µM (Fig. S2), the final concentration of maltotriose was set to 10 times higher than that value, or 60 µM in the case of competition.

### ITC

The thermodynamic parameters of the interactions between SPs0871 and maltotriose were determined using an iTC200 Microcalorimeter (Malvern Panalytical, Malvern, UK). Buffer conditions were 1 × PBS. The ITC cell was filled with 100 μM SPs0871 wild type or the W256A alanine mutant, and the ITC syringe was filled with 1 mM maltotriose. The thermodynamic parameters were calculated by fitting of the titration curve using ORIGIN 7.0 SR4 software (MicroCal, Northampton, MA, USA, ver7.0552, https://www.originlab.com/pdfs/O7_sr4_patch.pdf).

### Growth assay

JRS4 and SSI-1 were used as GAS strains. The medium used as well as the procedure up to the assay were the same as previously described^11^. All assays in CDM medium had a final concentration of 0.5% maltotetraose, 1% DMSO and 100 µM of the compound (95,186, 95186b, 95186c and 95186d). For SSI-1, an additional 0.1% glucose was added to the CDM medium containing maltotetraose. To confirm the bacteriostatic effect of compound 95,186, the bacterial culture incubated in CDM was inoculated into THY medium to achieve a final OD_600_ value of 0.01.

## Supplementary Information

Supplementary Information.

## Data Availability

The coordinates and structure factors of SPs0871 have been deposited in the PDB with entry code 9KHA. The datasets generated during and/or analyzed during the current study are available from the corresponding author upon reasonable request.
