# Supplementary material for: Development of a small compound that regulates the function of a maltodextrin-binding protein of Streptococcus pyogenes by multifaceted screenings
Source: Sci Rep. 2025 Jun 2;15:19341. doi: 10.1038/s41598-025-02175-9 (PMC12130509; doi:10.1038/s41598-025-02175-9)
Supplement: Supplementary file 1 — Supplementary Information. [file 41598_2025_2175_MOESM1_ESM.docx]

# ***Supporting Information***


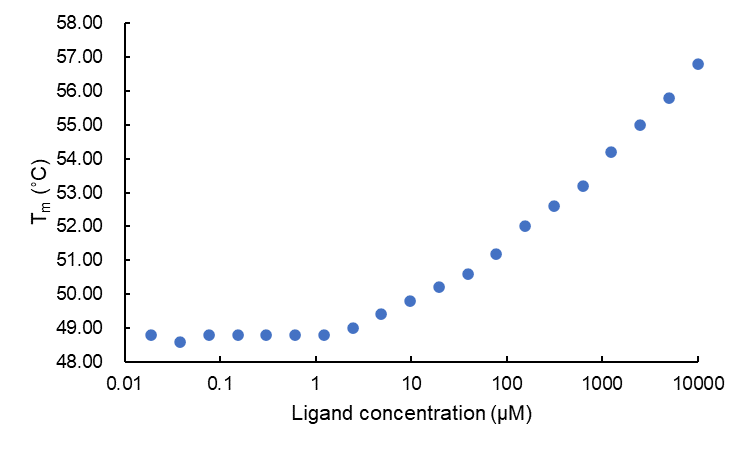


**Figure S1. Melting temperature of SPs0871 in the presence of various concentrations of maltotriose**


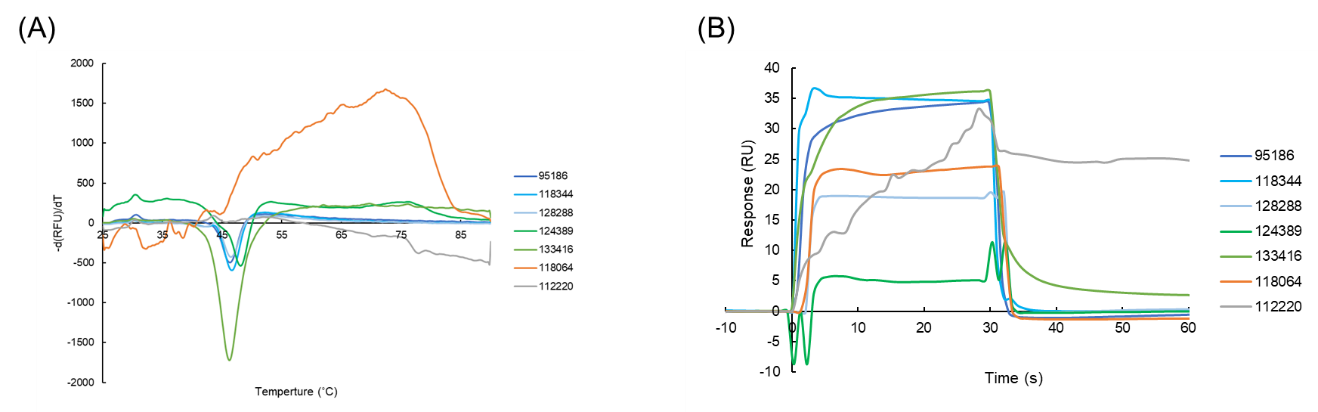


**Figure S2. Melting curve and SPR sensorgram of temperature for the seven compounds. (A) DSF screening. (B) SPR screening**
